# EGR1 mediates MDR1 transcriptional activity regulating gemcitabine resistance in pancreatic cancer

**DOI:** 10.1186/s12885-024-12005-2

**Published:** 2024-02-26

**Authors:** Zhe Yang, Feiran Chen, Dafu Wei, Fengping Chen, Haixing Jiang, Shanyu Qin

**Affiliations:** 1 Department of Gastroenterology, Guangxi Medical University Cancer Hospital, No 71 Hedi Road, Nanning, Guangxi Zhuang Autonomous Region PR China; 2 Department of Gastroenterology, The First Affiliated Hospital of Guangxi Medical University, No 6 Shuangyong Road, Nanning, Guangxi Zhuang Autonomous Region PR China

**Keywords:** EGR1, Pancreatic cancer, Gemcitabine resistance, MDR1

## Abstract

**Background:**

Gemcitabine is a cornerstone drug for the treatment of all stages of pancreatic cancer and can prolong the survival of patients with pancreatic cancer, but resistance to gemcitabine in pancreatic cancer patients hinders its efficacy. The overexpression of Early growth response 1(EGR1) in pancreatic ductal adenocarcinoma as a mechanism of gemcitabine chemoresistance in pancreatic cancer has not been explored. The major mechanisms of gemcitabine chemoresistance are related to drug uptake, metabolism, and action. One of the common causes of tumor multidrug resistance (MDR) to chemotherapy in cancer cells is that transporter proteins increase intracellular drug efflux and decrease drug concentrations by inducing anti-apoptotic mechanisms. It has been reported that gemcitabine binds to MDR1 with high affinity. The purpose of this research was to investigate the potential mechanisms by which EGR1 associates with MDR1 to regulate gemcitabine resistance in pancreatic cancer cells.

**Methods:**

The following in vitro and in vivo techniques were used in this research to explore the potential mechanisms by which EGR1 binds to MDR1 to regulate gemcitabine resistance in pancreatic cancer cells. Cell culture; in vitro and in vivo study of EGR1 function by loss of function analysis. Binding of EGR1 to the MDR1 promoter was detected using the ChIP assay. qRT-PCR, Western blot assays to detect protein and mRNA expression; use of Annexin V apoptosis detection assay to test apoptosis; CCK8, Edu assay to test cell proliferation viability. The animal model of pancreatic cancer subcutaneous allograft was constructed and the tumours were stained with hematoxylin eosin and Ki-67 expression was detected using immunohistochemistry.

**Findings:**

We revealed that EGR1 expression was increased in different pancreatic cancer cell lines compared to normal pancreatic ductal epithelial cells. Moreover, gemcitabine treatment induced upregulation of EGR1 expression in a dose- and time-dependent manner. EGR1 is significantly enriched in the MDR1 promoter sequence.Upon knockdown of EGR1, cell proliferation was impaired in CFPAC-1 and PANC-1 cell lines, apoptosis was enhanced and MDR1 expression was decreased, thereby partially reversing gemcitabine chemoresistance. In animal experiments, knockdown of EGR1 enhanced the inhibitory effect of gemcitabine on tumor growth compared with the sh-NC group.

**Conclusions:**

Our study suggests that EGR1 may be involved in the regulation of MDR1 to enhance gemcitabine resistance in pancreatic cancer cells. EGR1 could be a novel therapeutic target to overcome gemcitabine resistance in pancreatic cancer.

**Supplementary Information:**

The online version contains supplementary material available at 10.1186/s12885-024-12005-2.

## Introduction

Pancreatic ductal adenocarcinoma is a malignancy with a poor clinical prognosis, with a 10-year survival rate of only about 1% [1]. Gemcitabine has now become the standard of care for locally advanced and metastatic pancreatic cancer [2]. Unfortunately, progression of the disease is due to a complex phenomenon of high chemoresistance (both intrinsic and acquired), called multidrug resistance (MDR) [3], where treatment efficacy is hampered by resistance weeks after the initiation of therapy [4]. Therefore, the search for new influencing factors on chemoresistance in pancreatic cancer is important to improve treatment efficacy and predict drug response.

Early growth response genes 1(EGR1) is a nuclear protein that acts as a transcriptional regulator of a variety of genes, including tumor suppressor genes and oncogenes [5]. EGR1 plays a regulatory function in cell growth, but the role of EGR1 varies in different tumors [6]. High EGR1 expression has been found in a variety of cancers such as gastric, colorectal, hepatocellular, and cervical cancer and has been associated with poorer prognostic outcomes such as distant metastasis and poor survival [7–10]. On the other hand, EGR1 itself is a stress response protein whose expression can be upregulated when cells are exposed to drugs, growth factors, inflammatory factors, tumor necrosis factors, or other factors [11–13].

Aberrant induction of cell death pathways, overexpression of many different cellular pathways, transcription factors, and MDR1 are associated with resistance and sensitivity to gemcitabine [14, 15]. Overexpression of MDR1 results in acquired MDR due to their ability to transport multiple chemically diverse anticancer drugs [16–18]. Among them, ATP-binding cassette (ABC) transporter proteins such as MDR1,Breast cancer resistance protein (BCRP), and Multi-drug resistant associate protein 1(MRP1) are recognized molecules that promote the development of MDR [19] and can play an important role in gemcitabine chemoresistance in pancreatic cancer patients [20]. Moreover, it has been shown by molecular docking assays that gemcitabine has a high binding affinity to MDR1 [21]. It has also been reported that the efficacy window of gemcitabine associated with the function of efflux proteins such as MDR1 is concentration- and time-dependent [22].

EGR1 contains a highly conserved DNA-binding structural domain that binds to the consensus motif GCG(G/T)GGGCG [23], which can transactivate the expression of downstream target genes [24–26]. EGR1 is known to bind to GC elements on the proximal MDR1 promoter to enhance MDR1 transcription, thus controlling MDR1 expression at the transcriptional level. Furthermore, its site is functionally important for the regulation of the MDR1 gene, hence a potential therapeutic target [27, 28]. EGR1 has also been shown to mediate activation of the MDR1 promoter by the protein kinase C agonist TPA in hematopoietic cell lines [29]. In previous studies, EGR1 has been shown to bind to and act as a transcription factor for downstream target genes, but more functions of EGR1 have not been identified and the mechanism of action is not yet clear. In this research, we investigated the interaction between EGR1 and MDR1, hoping to bring new evidence for chemotherapy of pancreatic cancer.

## Materials and methods

### Cell cultures and reagents

The cell lines HPDE6-C7, CFPAC-1, BxPC-3, AsPC-1, and PANC-1 were purchased from the Shanghai Institute of Biochemistry and Cell Science, Chinese Academy of Sciences. Cells were cultured in a constant-temperature cell culture incubator at 37 °C with 5% CO_2_. HPDE6-C7 and PANC-1 were cultured in Gibco DMEM medium, BxPC-3 and AsPC-1 in Gibco 1640 medium, and CFPAC-1 cells in Gibco IMDM medium. The culture medium contained 10% fetal bovine serum and 1% penicillin and streptomycin. Gemcitabine was purchased from MCE (HY-B0003, MCE, New Jersey, USA).

### Stable transfection for EGR1 knockdown and plasmid overexpression of EGR1

The lentiviral shRNA for EGR1 (sh-EGR1) and the negative control scrambled shRNA (sh-Control) were designed and constructed by OBiO Technology (Shanghai) Corp., Ltd.The sequences of the shRNAs used in the research are in Supporting information (Additional file: Supplementary Table S1). Plasmids for EGR1 overexpression were supplied by MiaoLingPlasmid, and the plots are shown in the Supplementary Materials (Additional file: Supplementary Figure S1). The target cells were seeded into a 24well plate at 5 × 10^4^cells/well. When the cells were in good condition and the density reached 30–40%, the lentivirus was added for infection. After 12–16 h of virus infection, the medium in each experimental group was removed and replaced with fresh complete medium to continue the culture. The efficiency of lentivirus infection of target cells was determined by observation using fluorescence microscopy 72 h after virus infection. The efficiency of knockdown in stable cell lines was verified by qRT-PCR and Western blot.

### CCK8 assay

Cell proliferation was determined using the CCK8 assay (BS350, Biosharp, Anhui, China). Cells were seeded at a density of 5 × 10^3^ cells in 96-well plates with at least five replicate wells per group, then placed in a constant-temperature cell incubator at 37 °C with 5% CO_2_. After 24 h of incubation, gemcitabine was added. Then, after 48 h of incubation, CCK8 (10 µL) was added to each well and the cells were further incubated for 1 h prior to the assay. Absorbance value is 450 nm.Samples were taken in triplicate and the mean value was obtained.

### EdU assays

EdU assays were performed using the BeyoClick™ EdU Cell Proliferation Kit with Alexa Fluor 488 (C0071S, Beyotime, Shanghai, China). 5 × 10^4^ cells with or without transfection were seeded in 48-well plates. After 48 h of treatment (gemcitabine, 1 µmol/L), cells were incubated with EdU working solution for 2 h and then immunofluorescence staining was performed according to the manufacturer’s instructions.

### Western blot analysis

Total cellular proteins were prepared by lysing cells in RIPA buffer containing 1 mM phenylmethylsulfonyl fluoride (PMSF). After measuring their protein concentrations, cell lysates were separated equally on SDS-PAGE at 10–12% then transferred to polyvinylidene difluoride (PVDF) membranes (Millipore, Billerica, MA, USA) and blocked with 5% skim milk powder in TBST for 1 h. The membranes were incubated overnight at 4 °C with primary antibodies for EGR1 (22008-1-AP, ProteinTech, Wuhan, China, 1:3000), MDR1 (223336-1-AP, ProteinTech, 1:1000), and β-actin (66009-1-lg, ProteinTech, 1:20,000). Membranes were then washed and incubated with goat anti-mouse IgG (H + L) secondary antibody and fluorescent dye solution in 4× PEG (DyLight 800, SA5-35521, ThermoFisher, MA, USA) to probe the membranes. Finally, the grayscale values of the images were analyzed using ImageJ software, using β-actin as a loading control. The experiments were repeated three times and average values were obtained.

### Quantitative real-time polymerase chain reaction (qRT-PCR)

Total RNA from cells (HPDE6-C7, PANC-1, BxPC-3, AsPC-1, and CFPAC-1) was extracted using TRIZOL reagent (TaKaRa, Japan). RNA samples were reverse transcribed into cDNA using the PerfectStart Uni qPCR + RT kit (AUQ-01, TransGen Biotech Co, Beijing, China). The following primer sequences were used: EGR1 forward 5′-CAGCAGCAGCACCTTCAAC-3′, EGR1 reverse 5′-GTCTCCACCAGCACCTTCTC-3′,MDR1 forward 5′-ATCACCATCATCCCCCAGGA-3′,MDR1 reverse 5′-TGCAGTCCTCGAACTGTGTC-3′, GAPDH forward 5′-GCACCGTCAAGGCTGAGAAC-3′, and GAPDH reverse 5′-TGGTGAAGACGCCAGTGGA-3′. The expression of EGR1 was determined using qRT-PCR using Power SYBR Green PCR master mix (AUQ-01, TransGen Biotech Co, Beijing, China). Samples were run in triplicate and the mean values were obtained. The relative expression of mRNA was calculated using the 2^−ΔΔCT^ method, with GAPDH as the control.

### Annexin V apoptosis detection assay

Annexin V apoptosis detection assay was performed using the Annexin V-AF647/7-AAD fluorescent double-stained apoptosis detection kit (E-CK-A214, Elascience, Wuhan, China). 1 × 10^6^ cells in 1mL PBS were mixed, centrifuged at 1200 rpm for 5 min, then supernatant removed. Then, 100 µL of 1×Binding Buffer was added to each tube, resuspended, and 2.5 µL of Annexin V-AlexaFluor 647 and 2.5 µL of 7-AAD added. The solution was gently mixed and incubated for 15 min in the dark at room temperature (25 °C). Lastly, 200 µL of 1×Binding Buffer was added, and the CytoFLEX S flow cytometry system was used for detection within one hour. The experiment was repeated three times and average values were obtained.

### Chromatin immunoprecipitation (ChIP) assay

When the fusion of the target cells reached 70–80%, samples were obtained using the ChIP kit (CST9005, Boston, USA). First, to cross-link the proteins to the DNA, paraformaldehyde solution was added to the cells and left at room temperature for 10 min. After standing, glycine was added and mixed, and then the cells were incubated at room temperature for 5 min to stop cross-linking. Immediately thereafter, the cells are nucleated and chromatin sheared to obtain a sample of cross-linked chromatin fragments. Chromatin immunoprecipitation is performed by adding an equal amount of crosslinked chromatin samples per tube to the pre-made ChIP buffer, then chromosomes are eluted and de-crosslinked from the antibody/protein G microbeads, followed by centrifugation of the columns to obtain the purified DNA. qRT-PCR Reaction System is configured according to the instructions for the reagent and analyzed for qPCR ChIP enrichment efficiency. Prediction of EGR1 binding sites to promoter MDR1 using the JASPAR database(Additional file: Supplementary Table S2). The design sequences of the MDR1 primers are in the Supplementary file (Additional file: Supplementary Table S3).

### Immunohistochemical (IHC) analysis

Paraffin-embedded tissue samples were cut into 4-µm sections and dewaxed for IHC staining. The slides were then incubated with primary antibody Ki-67 (GB111499, Servicebio, Wuhan, China,1:1000) flat in a wet box overnight at 4 °C, and then incubated with secondary antibody(GB23303,Servicebio, Wuhan, China,1:200) for 50 min at 20 °C. After washing, freshly prepared DAB chromogenic solution (G1212, Servicebio, Wuhan, China) was added dropwise to the slides, and the color development time was controlled under the microscope. Then, the slides were restained with hematoxylin (G1004, Servicebio, Wuhan, China). Finally, the films were dehydrated and sealed.The results were interpreted under a white light microscope. Positive Ki-67 staining was assessed using an IHC score, which was calculated as follows: IHC score = percentage score × intensity score. The percentage of positive cells was divided into five classes (percentage score): (i) 0, < 10%; (ii) 1, 10–25%; (iii) 2, 25–50%; (iv) 3, 50–75%; and (v) 4, > 75%. In addition, the staining intensity was divided into four classes (intensity scores): (i) 0, no staining; (ii) 1, light brown; (iii) 2, brown; and (iv) 3, dark brown.

### Animal experiments

Female BALB/c nude mice at 4–6 weeks were purchased from the Animal Experiment Center of Guangxi Medical University to examine tumorigenicity.The nude mice were fed under the Specific Pathogen Free (SPF), 24–26 °C constant temperature environment.The cage equipment, bedding, drinking water and feed were disinfected and sterilized. Mice were randomly divided into 4 subgroups (*n* = 4 per group), and CFPAC-1/sh-Control vector and CFPAC-1/sh-EGR1 (2 × 10^6^ cells/mouse) transfected CFPAC-1 cells were inoculated subcutaneously into the right axilla of mice. The size of the tumors was measured weekly using vernier calipers 7 days after tumor cell injection, and the tumor volume was calculated using the formula: π/6 × L × W², where L is the length of the tumor and W is the width. After 7 days of tumor cell injection, when mice had tumor nodules with a palpable diameter of 3 mm or more, gemcitabine was administered twice a week at a dose of 10 mg/kg by intraperitoneal injection for 4 weeks. Control mice received saline, according to the same schedule.Euthanasia at the end of the experiment will be performed by intraperitoneal injection of sodium pentobarbital.Mice were executed 5 weeks after injection of tumor cells, and tumors were surgically removed, weighed, and analyzed by hematoxylin and eosin staining (H&E) and IHC.All experimental procedures were approved by the Medical Ethics Committee of the First Affiliated Hospital of Guangxi Medical University.

### Data collection from patient datasets

We extracted EGR1 expression data from two datasets, The Cancer Genome Atlas (TCGA) and Genotype-Tissue Expression (GTEx), for pancreatic cancer and normal pancreatic tissues. The datasets have been normalized for transcriptional base number per million mapped reads (TPM). Each expression value was logarithmically (TPM + 1) transformed. Prediction of EGR1 binding sites to promoter MDR1 using the JASPAR database. Data visualization was performed using the online tool available at the SangerBox 3.0 website.

### Statistical analysis

All statistical analyses were run in SPSS statistical software (version 25.0). Data are expressed as mean ± standard deviation (SD) of three independent experiments. All tests were two-sided, and *P* values < 0.05 were considered statistically significant. Graphs were generated using GraphPad Prism 8 software.

## Results

### The abnormal expression of EGR1 in pancreatic cancer cell lines

**Fig. 1:**
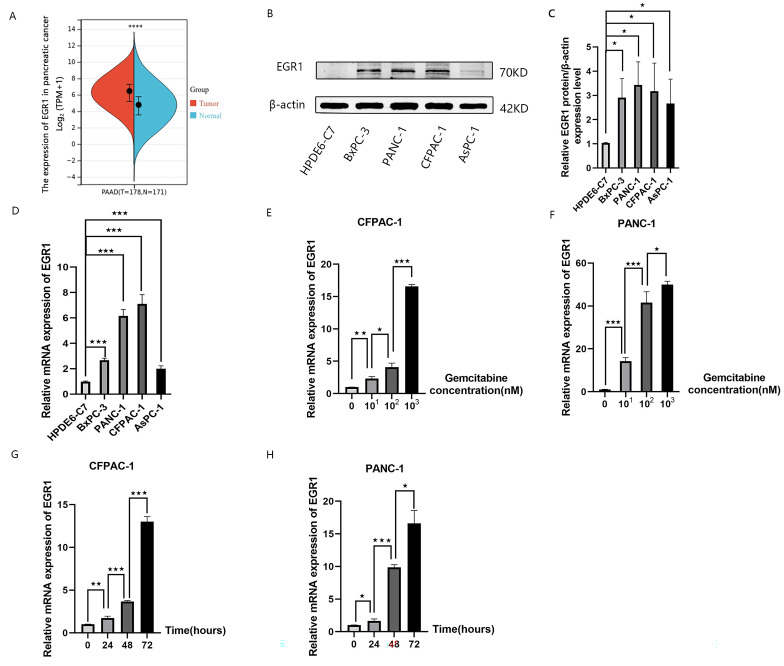
EGR1 expression in human pancreatic cancer cell lines. **(A)** Expression of EGR1 in pancreatic cancer and normal pancreatic tissues from The Cancer Genome Atlas (TCGA) and Genotype-Tissue Expression (GTEx) databases. **(B-D)** qRT-PCR and Western blot analysis showed higher expression of EGR1 in four pancreatic cancer cell lines (AsPC-1, BxPC-3, CFPAC-1, PANC-1) than in HPDE cells. Samples were from the same experiment and gels/blots were processed in parallel. (**E-F)** PANC-1 and CFPAC-1 cells were treated with different concentrations (0.01, 0.1, 1 µmol/L) of Gemcitabine for 48 h.**(G-H)** PANC-1 and CFPAC-1 cells were treated with 0.1 µmol/L concentration of Gemcitabine for 24, 48 and 72 h. The qRT-PCR analysis was performed to determine the expression levels of EGR1 mRNA. Data are expressed as mean ± SD. *P < 0.05; **P < 0.01; ***P < 0.001

Firstly, we assessed the expression of EGR1 in pancreatic cancer and normal pancreatic tissues from TCGA and GTEx databases, which showed that EGR1 expression levels were significantly higher in pancreatic cancer (Fig. 1A). Furthermore, we examined the expression of EGR1 protein and mRNA in human normal pancreatic ductal epithelial cells (HPDE6-C7) and four pancreatic cancer cell lines (BxPC-3, PANC-1, CFPAC-1, and AsPC-1) (Fig. 1B,C,and D). The results of both qRT-PCR and Western blot analysis showed that the expression of EGR1 was increased in different pancreatic cancer cell lines compared to normal pancreatic ductal epithelial cells.We detected the content of EGR1 in pancreatic cancer cells after gemcitabine intervention by qRT-PCR and found that gemcitabine intervention induced upregulation of EGR1 and the rise in expression increased with increasing dose and time (Fig. 1E,F,G,and H). PANC-1 and CFPAC-1 cells were treated with different concentrations (0.01, 0.1, 1 µmol/L) of gemcitabine for 48 h (Fig. 1E and F). PANC-1 and CFPAC-1 cells were treated with 0.1 µmol/L concentration of gemcitabine for 24, 48 and 72 h(Fig. 1G and H).Gemcitabine treatment induced the upregulation of EGR1 in a dose- and time-dependent manner.

### EGR1 mediates MDR1 transcriptional activity regulating gemcitabine resistance

**Fig. 2:**
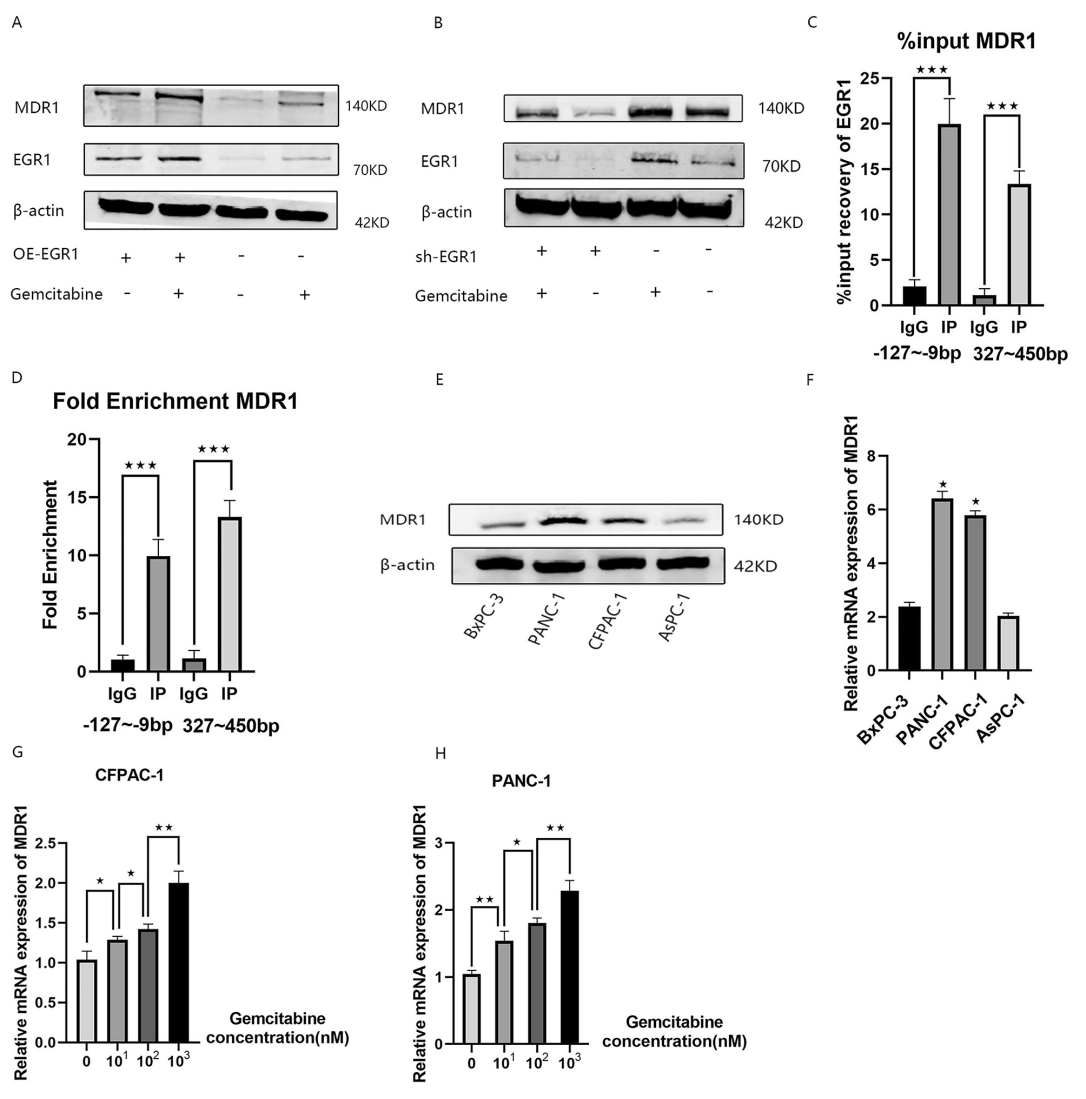
EGR1 mediates MDR1 transcriptional activity regulating gemcitabine resistance. (**A**) OE-EGR1 and OE-NC cells were treated with 0.1 µmol/L gemcitabine for 48 h. Changes in MDR1 and EGR1 were detected by Western blot analysis.(**B**) The sh-EGR1 cells and sh-NC cells were treated with 0.1 µmol/L gemcitabine for 48 h. Changes in MDR1 and EGR1 were detected by Western blot analysis. (C-D)Binding of EGR1 to the MDR1 promoter was detected using the ChIP assay. (**E-F**) Baseline expression of MDR1 in pancreatic cancer cell lines was detected by qRT-PCR and Western blot analysis.(G-H)Expression of MDR1 after gemcitabine treatment of CFPAC-1 and PANC-1 cells as detected by qRT-PCR.Samples were from the same experiment and gels/blots were processed in parallel.Data are expressed as mean ± SD. *P < 0.05; **P < 0.01; ***P < 0.001

**Fig. 3:**
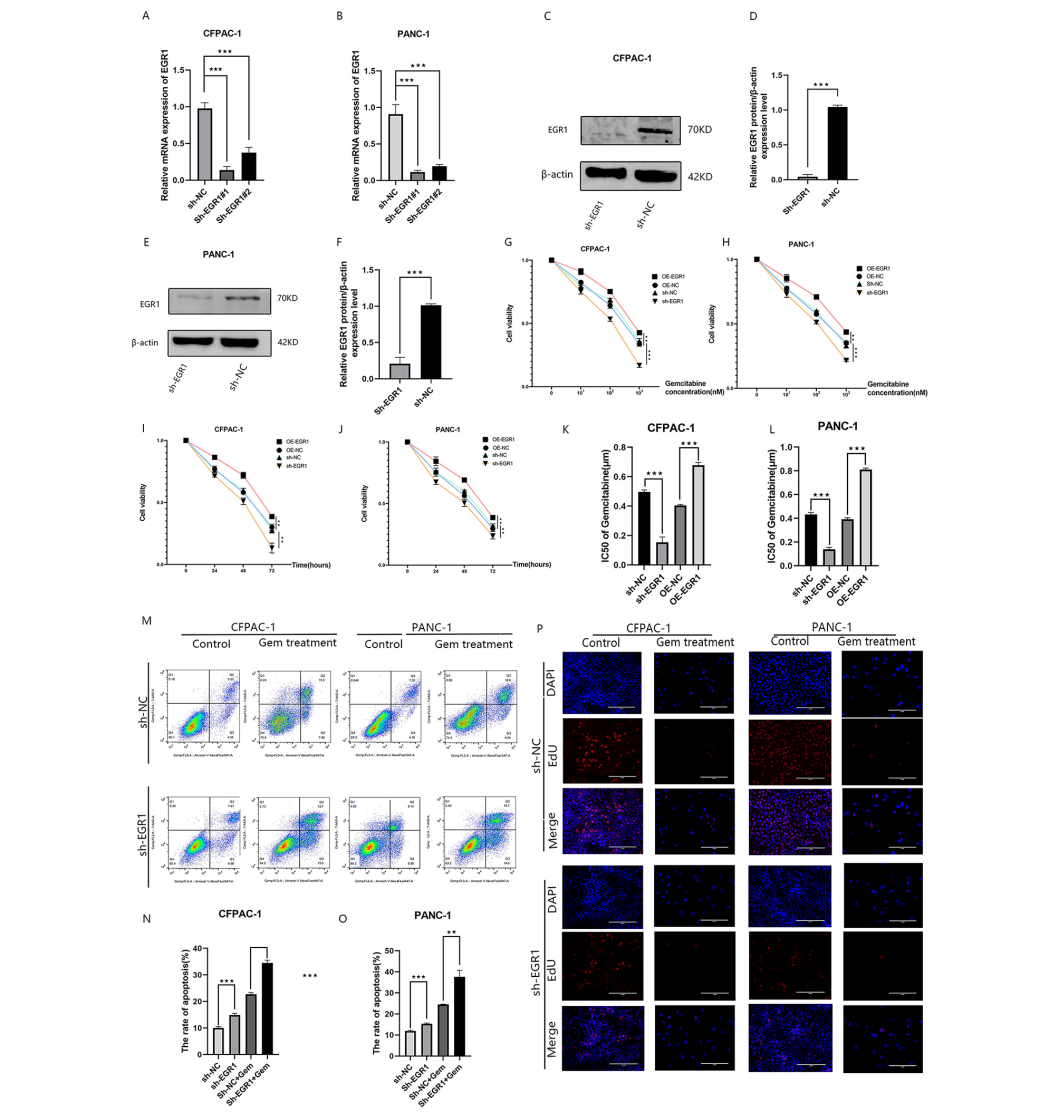
Interference with EGR1 expression affects the proliferative capacity of pancreatic cancer cells and can modulate cell sensitivity to Gemcitabine. (**A–F**) qRT-PCR and Western blot analysis showing the silencing efficiency of EGR1 in CFPAC-1 and PANC-1 cell lines. (**G-H**) Silenced or overexpressed CFPAC-1, and silenced or overexpressed PANC-1 cells were treated with different concentrations (0.01, 0.1, and 1 µmol/L) of gemcitabine for 48 h, and the cell viability was detected using the CCK8 assay. (**I-J**)Silenced or overexpressed CFPAC-1, silenced or overexpressed PANC-1 cells were treated with gemcitabine (0.1 µmol/L) for 24, 48, and 72 h, and cell viability was detected using the CCK8 assay.(**K-L**)CCK8 assay to detect IC50 in CFPAC-1 and PANC-1 cells silencing or overexpressing EGR1. (**M-O**) Flow cytometry of apoptosis of the indicated cells exposed to gemcitabine (1 µmol/L) for 48 h. (P)Representative fluorescent micrographs and quantification of EdU staining of the indicated cells after gemcitabine treatment (1 µmol/L) for 48 h.Scale bars: 200 μm.*P < 0.05; **P < 0.01; ***P < 0.001. Samples were from the same experiment and gels/blots were processed in parallel. β-actin was used as a loading control.

OE-EGR1 and OE-NC cells were treated with 0.1 µmol/L gemcitabine for 48 h. Changes in MDR1 and EGR1 were detected by Western blot analysis. The expression of MDR1 and EGR1 increased after Gemcitabine treatment, as detected by Western blot analysis, and MDR1 expression was positively correlated with EGR1(Fig. 2A). The sh-EGR1 cells and sh-NC cells were treated with 0.1 µmol/L gemcitabine for 48 h. Changes in MDR1 and EGR1 were detected by Western blot analysis.After silencing the expression of EGR1, the expression of MDR1 decreased compared to the sh-NC group(Fig. 2B).As verified by the CHIP assay, EGR1 is significantly enriched in the MDR1 promoter sequence(Fig. 2C and D).Subsequently, we examined the expression of MDR1 in pancreatic cancer cell lines by Western blot analysis and qRT-PCR, and found that the expression in CFPAC-1 and PANC-1 cell lines was higher than that in other pancreatic cancer cell lines(Fig. 2E and F).The use of gemcitabine dose-dependently increased MDR1 expression in CFPAC-1 and PANC-1 cell lines(Fig. 2G and H).To further investigate the functional role of EGR1 in pancreatic cancer cells, two shRNA knockdown vectors for EGR1 were constructed and transfected into CFPAC-1 and PANC-1 cells to stably reduce the expression of EGR1. qRT-PCR and Western blot analysis showed changes in mRNA and protein levels, respectively (Fig. 3A,B,C,D,E,and F). To assess the effect of EGR1 on cell survival and gemcitabine sensitivity, we first silenced or overexpressed EGR1 expression, and then treated the cells with gemcitabine at different doses and for different durations, which were assayed using the CCK8 assay(Fig. 3G,H,I,and J).Treatment of silenced or overexpressed CFPAC-1 cells and silenced or overexpressed PANC-1 cells with different concentrations (0.01, 0.1, and 1 µmol/L) of gemcitabine for 48 h revealed that the viability of cells that silenced EGR1 decreased, while that of cells that overexpressed EGR1 increased.(Fig. 3G and H). After treatment of silenced or overexpressed CFPAC-1, and silenced or overexpressed PANC-1 cells with gemcitabine (0.1 µmol/L) for 24, 48, and 72 h, the viability of cells with silenced EGR1 was decreased, and the viability of cells overexpressing EGR1 was increased.(Fig. 3I and J). we found that the value of IC50 became lower by silencing EGR1 expression and elevated by overexpressing EGR1(Fig. 3K and L).Then, we further investigated whether EGR1 affects Gemcitabine-induced apoptosis in pancreatic cancer cells. For this purpose, we performed Annexin V apoptosis detection assay (Fig. 3M,N,and O).The results showed that after interfering with EGR1 expression in CFPAC-1 and PANC-1 cells, apoptotic cells were significantly increased after knockdown of EGR1 compared with sh-NC.The EdU assay showed a decrease in the proliferative capacity of pancreatic cancer cells after silencing EGR1 expression(Fig. 3P).

### EGR1 knockdown enhances the sensitivity of pancreatic cancer cells to gemcitabine-induced growth in vivo

**Fig. 4:**
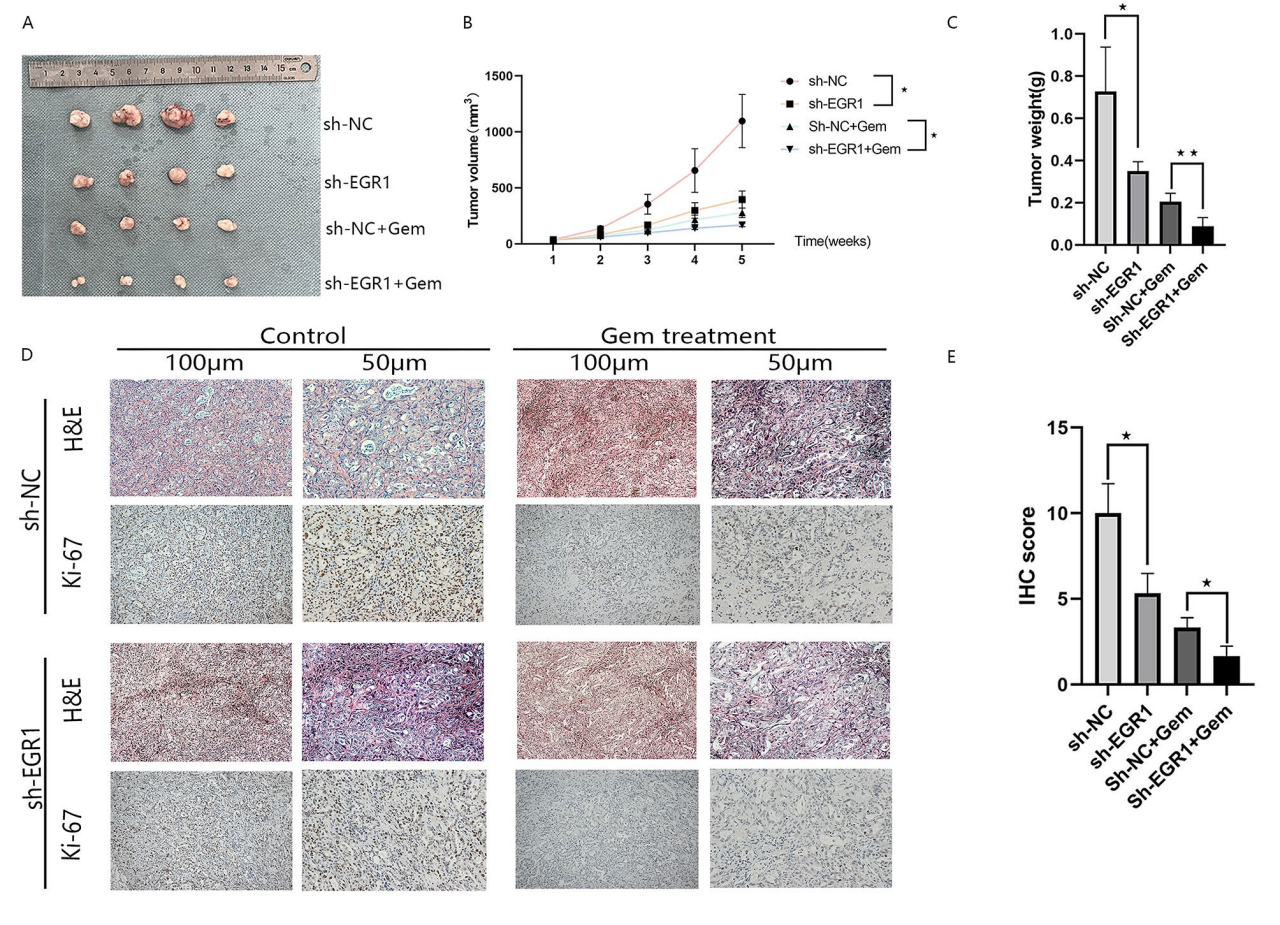
EGR1 knockdown increases the sensitivity of pancreatic cancer cells to gemcitabine-induced growth in vivo. (**A**) Representative photographs of excised tumors from different groups of BALB/C nude mice are shown. (**B**) Growth curves showing changes in tumor volume in different groups of mice; tumor growth was assessed once a week starting from the injection of gemcitabine (10 mg/kg). (**C**) The weight of tumors removed from each group of mice. (**D**) Representative H&E-stained and immunohistochemical images. Scale bar: 50 and 100 μm. (**E**) Positive Ki-67 staining was assessed using IHC score. Data are expressed as mean ± SD. *P < 0.05; **P < 0.01; ***P < 0.001

To further assess the sensitivity of EGR1 to gemcitabine in vivo, CFPAC-1 cells stably expressing sh-NC or sh-EGR1 were subcutaneously xenografted in the right axilla of 4–6-week-old BALB/c female nude mice and treated with 10 mg/kg gemcitabine. Perform twice weekly intraperitoneal injections of gemcitabine,after 4 weeks of treatment(Fig. 4A). The tumor tissues of nude mice co-treated with knockdown EGR1 and gemcitabine showed decreased tumor volume, decreased body weight, and lower proliferation capacity than the sh-NC group, enhancing the inhibitory effect of gemcitabine on tumor growth (Fig. 4B and C). Ki-67 is a proliferating cell-associated nuclear antigen whose function is closely related to mitosis and can be used as a marker of cell proliferation. It is expressed in the G1, S, G2 and M phases of the cell cycle, but not in the G0 phase, and is closely related to the degree of differentiation, infiltration, metastasis and prognosis of many tumors. Due to the short half-life, it can accurately reflect the proliferative activity of cells. In our immunohistochemical results, after knockdown of EGR1, Ki-67 positivity in animal tumor tissues was reduced compared with that in the sh-NC group, indicating that tumor proliferation and growth were slowed down(Fig. 4D and E).

## Discussion

Gemcitabine is the cornerstone drug used in the treatment of pancreatic cancer and has become the standard of care for locally advanced and metastatic pancreatic cancer [30]. However, its therapeutic efficacy is ultimately hampered by drug resistance [31]. Therefore, understanding the molecular mechanisms of gemcitabine resistance is essential to improve treatment strategies for pancreatic cancer.Several reports have confirmed that EGR1, as a transcription factor [32], is involved in regulating a variety of cellular processes, including proliferation, differentiation, angiogenesis, apoptosis, tumor invasion, and metastasis [6, 33]. Moreover, EGR1 is aberrantly expressed in different types of cancers and is involved in their development [34–36].

In this study, we found that EGR1 expression is elevated in pancreatic adenocarcinoma. Therefore, we focused on the role of EGR1 in pancreatic adenocarcinoma by observing the effects produced by silencing this gene in vitro and in vivo. Notably, EGR1 silencing inhibited pancreatic cancer cell growth and promoted apoptosis.The conclusion that EGR1 expression may affect the sensitivity of pancreatic cancer to gemcitabine provides a new solution for the clinical reduction of drug resistance in pancreatic cancer. In other findings, knockdown of EGR1 using EGR1 siRNA enhanced the growth and chemosensitivity of breast cancer cell lines to capsaicin [37].It is now reported that EGR1 can promote pancreatic cancer migration and invasion by controlling the expression of SNAI2 and modulating the EMT pathway [38]. In prostate cancer, siRNA downregulation of EGR1 expression inhibited the growth of the human prostate cancer cell line PC-3 [39]. Nonetheless, these results need to be further investigated on a larger scale to demonstrate that EGR1 expression can be used as a valid biomarker to evaluate treatment progression in patients with pancreatic cancer.

In patients with advanced pancreatic cancer, frequent expression of MDR1 may adversely affect the sensitivity and benefit of chemotherapy [40]. MDR1 induces cancer cells to acquire chemoresistance by affecting the efflux of anticancer drugs from cells and decreasing intracellular drug concentrations [41]. Previous reports have illustrated that ABC transporter protein-mediated reversal of MDR may be due to downregulation of ABC protein expression or altered subcellular localization [19]. EGR1 is an important transcription factor that specifically recognizes and binds target genes, regulating their transcription and causing a variety of pathophysiological responses [33]. The results showed that the expression of EGR1 was positively correlated with the expression of the multidrug resistance gene MDR1.In this research, we used the JASPAR database to predict that EGR1 has multiple binding sites to the MDR1 promoter sequence, and found that EGR1 was significantly enriched in the MDR1 promoter sequence by CHIP assay, and that EGR1 enhances the transcription of MDR1 by regulating the expression of MDR1 at the transcriptional level, thereby affecting the sensitivity of pancreatic cancer cells to gemcitabine, which provides evidence that EGR1 mediates the regulation of the resistance of pancreatic cancer cells to gemcitabine by MDR1.

In summary, EGR1 knockdown promotes apoptosis and inhibits gemcitabine resistance in pancreatic cancer cells. Our study shows for the first time that EGR1/MDR1 axis activation promotes gemcitabine resistance in pancreatic cancer by inhibiting apoptosis.The findings demonstrate that downregulation of EGR1 expression has clinical benefits for pancreatic cancer treatment and may be a potential target for gemcitabine chemoresistance in pancreatic cancer. The role and mechanism of EGR1 in pancreatic cancer cell lines can be further explored in the future, providing a new angle for the development of strategies for selective modulators of gene expression.

## Conclusions

In conclusions, this research suggests that EGR1-mediated upregulation of MDR1 play a crucial role in the induction of gemcitabine resistance in pancreatic cancer cells.EGR1 is a nuclear protein that acts as a transcriptional regulator of a variety of genes, including tumor suppressor genes and oncogenes. Targeting EGR1 may be a novel therapeutic strategy for pancreatic cancer to overcome gemcitabine resistance mediated by MDR1.

### Electronic supplementary material

Below is the link to the electronic supplementary material.

Supplementary Material 1

Supplementary Material 2

Supplementary Material 3

Supplementary Material 4

Supplementary Material 5

## Data Availability

The datasets generated or analyzed in this research are available from open databases. In this study, we used the following databases for data collection, result analysis and visualization: NCBI(https://www.ncbi.nlm.nih.gov/),TCGA ( https://portal.gdc.cancer.gov/),SangerBox 3.0 (http://vip.sangerbox.com/),JASPAR (https://jaspar.genereg.net/).

## Abbreviations

EGR1: Early growth response genes 1
MDR: Multidrug resistance
ABC: ATP-binding cassette
MRP1: Multi-drug resistant associate protein 1
BCRP: Breast cancer resistance protein
TCGA: The Cancer Genome Atlas
GTEx: Genotype-Tissue Expression databases
