# Supplementary material for: EGR1 mediates MDR1 transcriptional activity regulating gemcitabine resistance in pancreatic cancer
Source: BMC Cancer. 2024 Feb 26;24:268. doi: 10.1186/s12885-024-12005-2 (PMC10895816; doi:10.1186/s12885-024-12005-2)
Supplement: Supplementary file 1 — Supplementary Material 1 [file 12885_2024_12005_MOESM1_ESM.pdf]

Supplementary Table S1. The shRNA sequences.

Maker	Gene	Gene ID	TargetSeq
Y22721	EGR1	NM_001964.3	CGGTTACTACCTCTTATCCAT
Y22722	EGR1	NM_001964.3	CTGTCTACTATTAAGGCCTTT
Negative control			CCTAAGGTTAAGTCGCCCTCG
