# Supplementary figures and images for: EGR1 mediates MDR1 transcriptional activity regulating gemcitabine resistance in pancreatic cancer
Source: BMC Cancer. 2024 Feb 26;24:268. doi: 10.1186/s12885-024-12005-2 (PMC10895816; doi:10.1186/s12885-024-12005-2)

Supplementary Figure S1. Plasmid mapping for overexpression of EGR1.

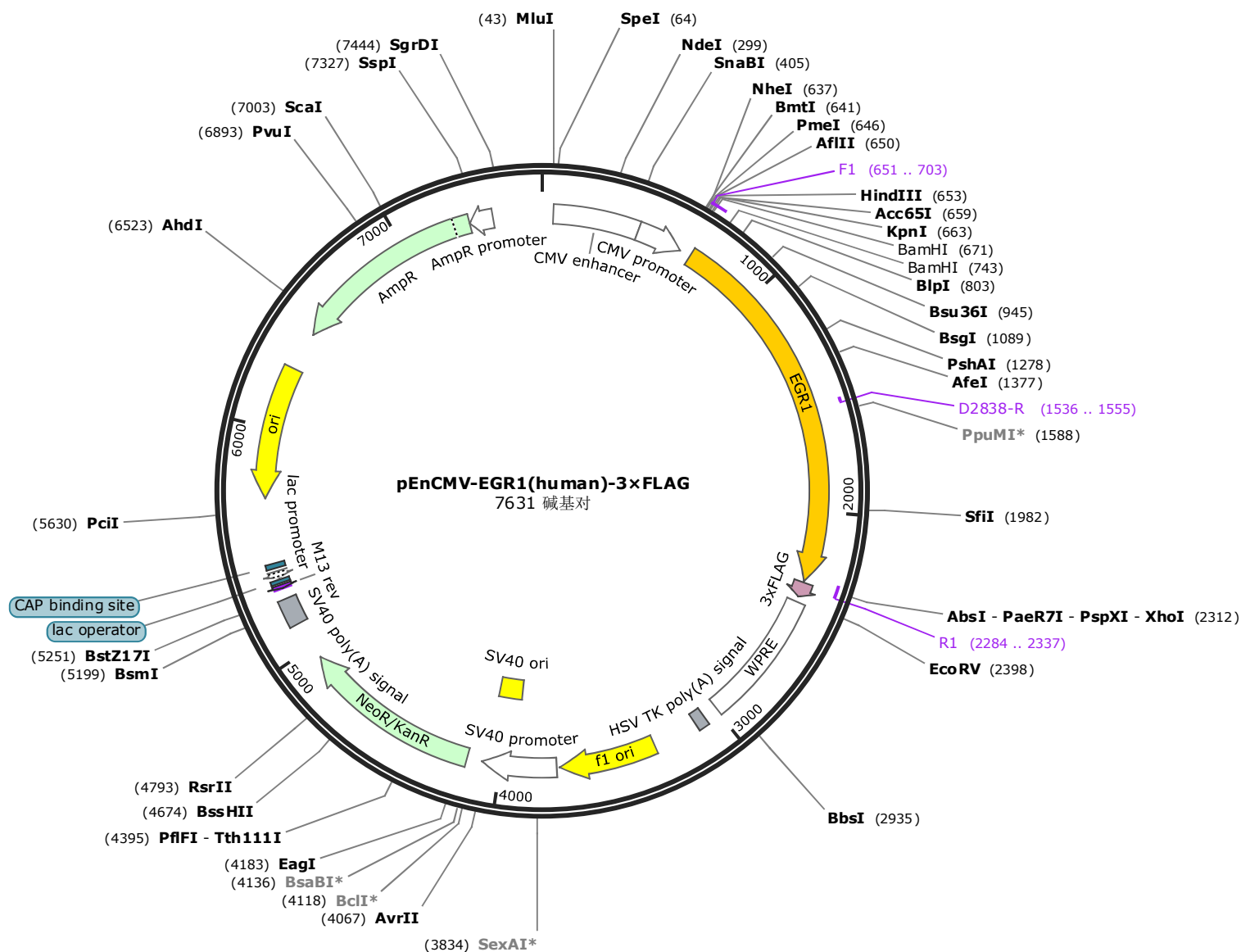

Supplement: Supplementary file 2 — Supplementary Material 2 [file 12885_2024_12005_MOESM2_ESM.pdf]
