# Supplementary material for: EGR1 mediates MDR1 transcriptional activity regulating gemcitabine resistance in pancreatic cancer
Source: BMC Cancer. 2024 Feb 26;24:268. doi: 10.1186/s12885-024-12005-2 (PMC10895816; doi:10.1186/s12885-024-12005-2)

Supplementary Figure S2. Original image of intact gels/blots without cropping.Thebands were visualized using the Odyssey Infrared Imaging System.Samples were from the same experiment and gels/blots were processed in parallel.

Figure 1B:

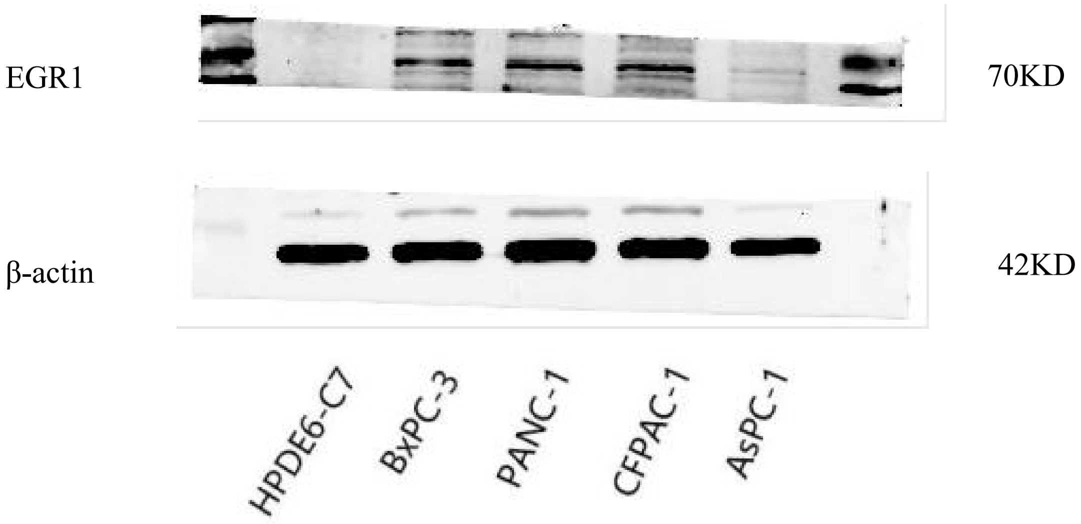

Figure 2A:

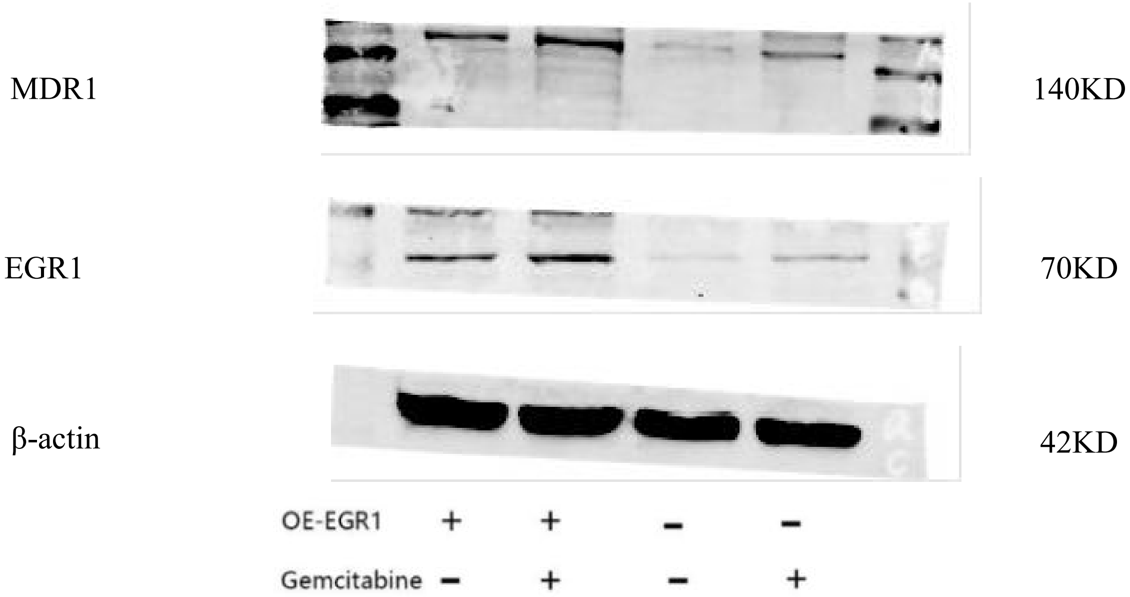

Figure 2B:

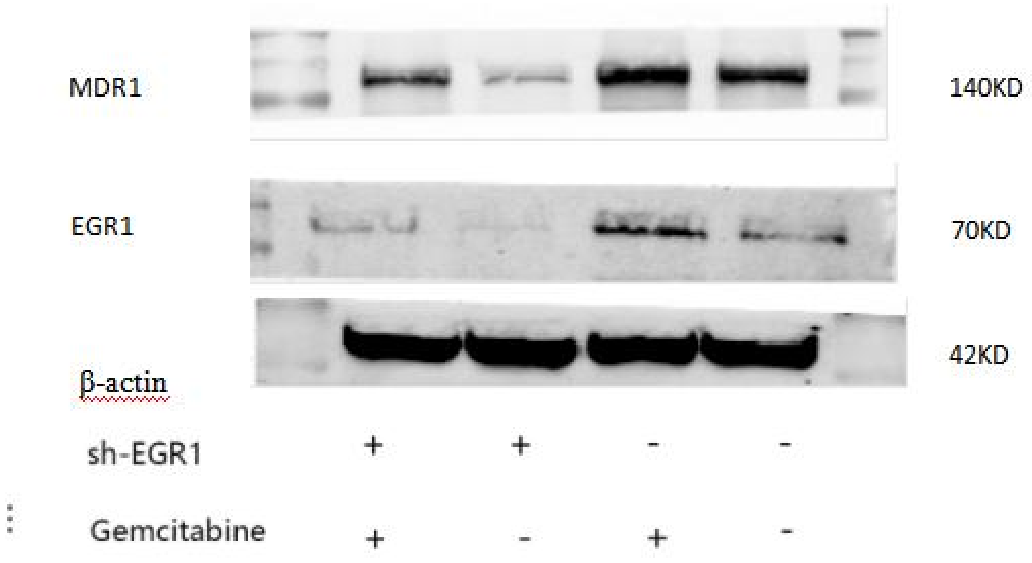

Figure 2E

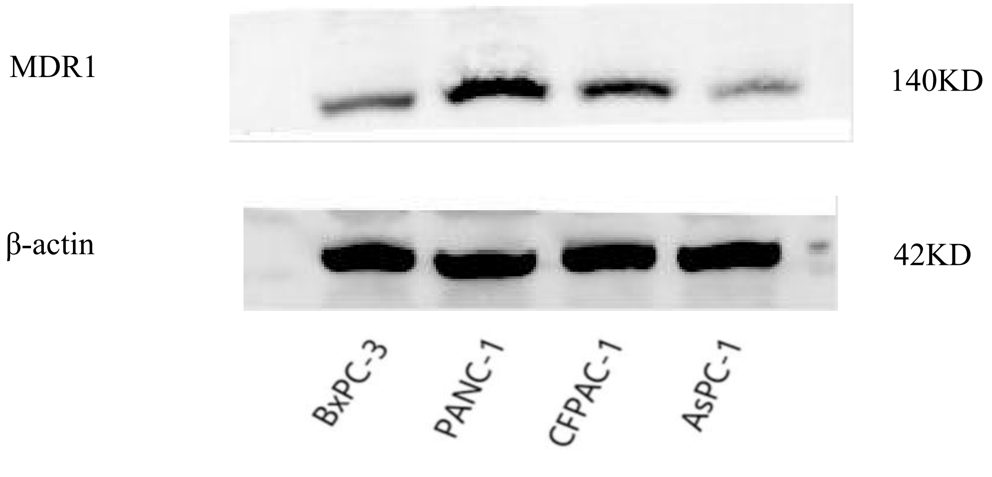

Figure 3C:

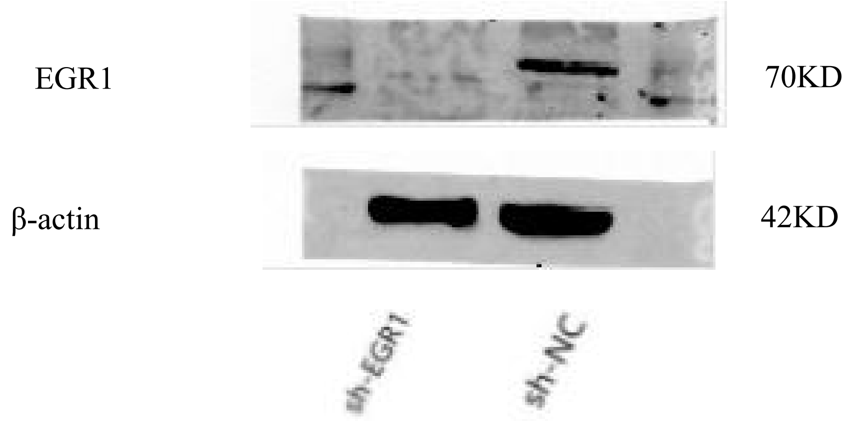

Figure 3E:

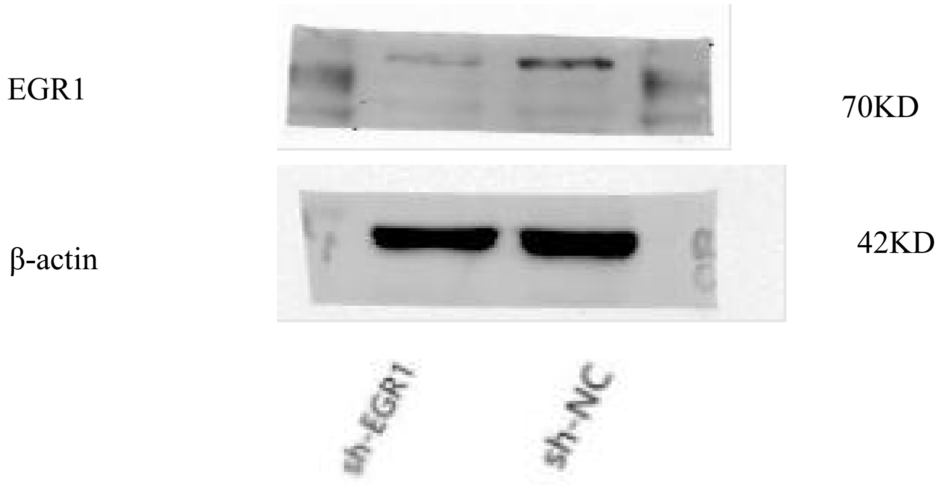

Supplement: Supplementary file 3 — Supplementary Material 3 [file 12885_2024_12005_MOESM3_ESM.pdf]
