# Supplementary material for: EGR1 mediates MDR1 transcriptional activity regulating gemcitabine resistance in pancreatic cancer
Source: BMC Cancer. 2024 Feb 26;24:268. doi: 10.1186/s12885-024-12005-2 (PMC10895816; doi:10.1186/s12885-024-12005-2)
Supplement: Supplementary file 4 — Supplementary Material 4 [file 12885_2024_12005_MOESM4_ESM.pdf]

Supplementary Table S2. Binding sites predicted using the JASPAR database.

Matrix ID	Name	Score	Relative score	Sequence ID	Start	End	Strand	Predicted sequence
MA0162.2	MA0162.2.EGR1	11.8686	0.907282795	MDR1	1947	1960	-	GCTCAGCCCACGCC
MA0162.4	MA0162.4.EGR1	11.931647	0.868399778	MDR1	1946	1959	-	CTCAGCCCACGCCC
MA0162.2	MA0162.2.EGR1	7.582126	0.861378251	MDR1	734	747	-	CTCCCGCCACCACG
MA0162.4	MA0162.4.EGR1	11.396373	0.859039109	MDR1	1940	1953	-	CCACGCCCCGGCGC
MA0162.2	MA0162.2.EGR1	7.293762	0.858290115	MDR1	1941	1954	-	CCCACGCCCCGGCG
MA0162.1	MA0162.1.Egr1	9.876793	0.855590951	MDR1	2400	2410	+	TGGGTGGGAGG
MA0162.2	MA0162.2.EGR1	7.022378	0.855383817	MDR1	2452	2465	-	TGCCCGCCGCCAGT
MA0162.2	MA0162.2.EGR1	6.713896	0.852080231	MDR1	637	650	-	CGCCCGCCTCGGCC
MA0162.2	MA0162.2.EGR1	6.5903635	0.850757304	MDR1	2400	2413	-	CTTCCTCCCACCCA
MA0162.1	MA0162.1.Egr1	9.552036	0.847415311	MDR1	1947	1957	+	GGCGTGGGCTG
MA0162.2	MA0162.2.EGR1	6.0204873	0.844654407	MDR1	2396	2409	-	CTCCCACCCACCGC
MA0162.3	MA0162.3.EGR1	9.317801	0.841155515	MDR1	1939	1952	-	CACGCCCCGGCGCT
MA0162.2	MA0162.2.EGR1	5.6225944	0.840393307	MDR1	2389	2402	-	CCACCGCCCGCGGG
MA0162.2	MA0162.2.EGR1	5.430656	0.838337807	MDR1	728	741	-	CCACCACGCCCGGC
MA0162.1	MA0162.1.Egr1	9.179453	0.838035647	MDR1	1941	1951	+	CGCCGGGGCGT
MA0162.4	MA0162.4.EGR1	9.729894	0.829896382	MDR1	2399	2412	-	TTCCTCCCACCCAC
MA0162.4	MA0162.4.EGR1	9.4916935	0.825730832	MDR1	2388	2401	-	CACCGCCCGCGGGC
MA0162.4	MA0162.4.EGR1	9.350056	0.823253926	MDR1	2395	2408	-	TCCCACCCACCGCC
MA0162.2	MA0162.2.EGR1	3.9941475	0.822954005	MDR1	2209	2222	-	GCCCTTCTCCCGTG
MA0162.2	MA0162.2.EGR1	3.9325664	0.822294523	MDR1	634	647	-	CCGCCTCGGCCTCC
MA0162.1	MA0162.1.Egr1	8.546276	0.822095632	MDR1	844	854	+	AGCCTGGGCGA
MA0162.1	MA0162.1.Egr1	8.468084	0.82012718	MDR1	2389	2399	+	CCCGCGGGCGG
MA0162.2	MA0162.2.EGR1	3.5798502	0.818517227	MDR1	797	810	-	GCTCCGCCTCCCGG
MA0162.2	MA0162.2.EGR1	3.4573984	0.817205871	MDR1	2438	2451	-	GCGATTCTCCCTCC
MA0162.3	MA0162.3.EGR1	7.2014327	0.815927338	MDR1	1945	1958	-	TCAGCCCACGCCCC
MA0162.2	MA0162.2.EGR1	2.999795	0.812305322	MDR1	771	784	-	CTCCTGCCTCAGCC
MA0162.2	MA0162.2.EGR1	2.9170768	0.81141948	MDR1	2125	2138	-	GCCCCTACCTCGCG
MA0162.2	MA0162.2.EGR1	2.513984	0.807102693	MDR1	768	781	-	CTGCCTCAGCCTCC
MA0162.2	MA0162.2.EGR1	2.4688914	0.806619789	MDR1	2392	2405	-	CACCCACCGCCCGC
MA0162.2	MA0162.2.EGR1	2.4264252	0.806165012	MDR1	1893	1906	-	TGACTGCTCCCGGC
MA0162.2	MA0162.2.EGR1	2.3207533	0.805033355	MDR1	1626	1639	-	ACTCCGACCTCTCC
MA0162.2	MA0162.2.EGR1	2.0291958	0.801911017	MDR1	2353	2366	+	GCACAGCCCGCGCG
MA0162.2	MA0162.2.EGR1	1.9083457	0.800616814	MDR1	602	615	+	CTGTAGCTCACGCC
