# Supplementary material for: EGR1 mediates MDR1 transcriptional activity regulating gemcitabine resistance in pancreatic cancer
Source: BMC Cancer. 2024 Feb 26;24:268. doi: 10.1186/s12885-024-12005-2 (PMC10895816; doi:10.1186/s12885-024-12005-2)
Supplement: Supplementary file 5 — Supplementary Material 5 [file 12885_2024_12005_MOESM5_ESM.pdf]

Supplementary Table S3. MDR1 primer design sequence

Primer size	Primer name	sequences
118	human MDR1 ChIP F-primer (-127~-9)	CAGCATTCAGTCAATCCGGG
	human MDR1 ChIP R-primer (-127~-9)	CTTCCTGTGGCAAAGAGAGC
123	human MDR1 ChIP F-primer (327~450)	GCACTGGACCATGTTGCC
	human MDR1 ChIP R-primer (327~450)	GATTCTCCCTCCCGGTTCC
